# Supplementary material for: Methods for a Non-Targeted Qualitative Analysis and Quantification of Benzene, Toluene, and Xylenes by Gas Chromatography-Mass Spectrometry of E-Liquids and Aerosols in Commercially Available Electronic Cigarettes in Mexico
Source: Int J Environ Res Public Health. 2024 Sep 30;21(10):1308. doi: 10.3390/ijerph21101308 (PMC11507580; doi:10.3390/ijerph21101308)
Supplement: Supplementary file 1 [file ijerph-21-01308-s001.zip › ijerph-3090453-supplementary.pdf]

Supporting Information

Article

Methods for a non-targeted qualitative analysis and quantification of benzene, toluene and xylenes by gas chromatography-mass spectrometry of e-liquids and aerosols in commercially available electronic cigarettes in Mexico.

1. Methodology for e-liquids analysis

Equipment:
Gas chromatograph GC-MS, Agilent Instrument, equipped with an EI source and a 7890A GC / 7890B system, Agilent, detector MSD 5975C and 5977A (Agilent Technologies, Santa Clara, CA, USA) was employed for the analysis. Software used was Environmental ChemStation, MSD CHEMSTATION version E.02.00493 and the library comparison data used was NIST08L. Column type DB-624 J&W Scientific 30m x 320 mm x 1.8 µm and also 2B-5MS plus 60mm x0.25 mm ID x 0.25 µm.
Materials and reagents
Helium was used as carrier gas at a constant flow.
Karal brand methanol (HPLC quality) and ultrapure water were purchased and used for the extractions and analysis.

Non-targeted

The used software was Environmental ChemStation, MSD ChemStation version E.02.00493, Agilent Technologies. For the Qualitative Analysis, NIST Library NIST08.L was used, and match/quality of identification was filtered on printed files to 70%.

Sample preparation

The cartridge, whether liquid or absorbent material containing the e-liquid, was extracted either with ultrapure water or methanol. Individual stock extracts of e-liquids were obtained by 30-minute sonication of the inner cartridges and stored in a closed glass vial at 4°C.For sampling, 500 mL were taken and diluted in organic free water to 50mL. The injection into the chromatography equipment was carried out through the purge and trap module at a temperature of 40oC for 30 min, injection volume 1 mL.

Chromatographic conditions:

Flow: 1.22 mL/min

Detection: The ionization source and the quadrupole were maintained at 230°C and 150°C, respectively.

Temperature: Oven was maintained at 40 °C for 5 min, then a gradient of 8 °C /min at 240 °C during 1 min was used.

Injection sample of 1µL, split ratio of 15:1, split flow of 18.3 ml/min.

BTX Quantification

For the quantification of BTX (volatile organic compounds), a BTEX standard 2000 μg/L, P&T Methanol, 1mL/ampul, Restek, and an internal standard cat. 30213 concentration 2000 μg/L was used. Calibration curve of eight levels, concentrations 0.5, 1.0, 1.5,2.5, 4.0, 5.0, 10 y 20 ppb were achieved. Eight levels were injected for triplicate. R^2 values ≥ 0.98, and at least five(xylenes), six (benzene), and seven (toluene) points were the acceptation criteria. Analytes were monitored in SCAN mode.

2. Methodology for aerosols analysis

Equipment: Gas chromatograph Agilent, 7890B, coupled to a mass quadrupole spectrometer Agilent, 5977A. Thermal desorption unit MARKES unity 2, autosampler MARKES Ultra TD. Tube charger, MARKES, TC-20 and vacuum pump SKC model 224-PCXR8 was used for direct sampling of the aerosols, and chromatographic column Phenomenex ZB-5MSplus 60m x 0.25 mm I.D. X 0.25μm.

Materials and reagents: Nitrogen gas 5.0 ultra-high purity 99.999% (thermal desorption and sampling), Helium gas 5.0 ultra-high purity 99.999% (GC), Methanol HPLC grade, TEDIA brand. Sample tubes, MARKES, ¼" x 3 ½", packed with sorbent Tenax TA for aerosol sampling.

Aerosol blank was treated at the same analysis conditions, using a nitrogen flux of 1L/min during 2 seconds into the Tenax tube, using the vacuum pump and a PTFE tube.

Software: Acquisition and instrumental control of the GC, MassHunter Data Analysis GC/MS version B.07.02.1938 Agilent Technologies.

Compounds associated to the column bleed, like siloxanes were discarded of the samples and blanks.

Substances contained in the blanks were eliminated from samples by subtraction.

Non-targeted

The used software was Environmental ChemStation, MSD ChemStation version F.01.00.1903, Agilent Technologies. For the unknown analysis, MassHunter WorkStation Software Quantitative Analysis B.07.01/Build 7.1.524.0 Unknows Analysis, Agilent Technologies.

Each e-cigarette device was connected to the vacuum pump (250 mL/min), using a PTFE tube and a nitrogen flux (1L/min, during 2 sec). The sample consisted in two puffs, taken after the fifth puff.

Desorption method: A temperature of 325°C, for 15 minutes, using a trap flow of 20 mL/min and trap temperature of -30°C was programmed.

Chromatographic conditions:

Flow: 1 mL/min

Detection: The ionization source and the quadrupole were maintained at 230°C and 150°C, respectively.

Temperature: Oven was maintained at 35ºC for 3 min, then a gradient of 4 ºC/min, at 70 ºC, for 7 min; then a 6 ºC/min at 120 ºC for 7 min; then a rate of 10 ºC/min at 150 ºC for 7 min; then 15 ºC/min at 250 ºC for min; at least 20 ºC/min at 310 ºC for 5 min.

Injection sample of 1µL, split ratio of 32:1, split flow of 32 ml/min.

For the qualitative analysis, compounds having less than 70% match were discarded, having concentrations lower than first level toluene curve (10.0045 ng), and libraries NIST11.L, 525_DRS.L, 8270_DRS.L, CF_SOLV.L and CP_SOLV.L were used.

Note: In the case of ethylbenzene probably present in the samples, in some cases the MS signal was not aligned with primary ion, and no coincidence on the abundance % was observed. This is due to an interference signal at the same retention time than ethylbenzene, sharing ions with the compound, increasing the concentration and the abundance %, changing the corresponding retention time. For this samples, a concentration below 10 ng (1st level curve) will be appropriate.

BTX Quantification

Software: For the processing and quantitative analysis, MassHunter Worstation Software, Quantitative Analysis Version B.06.00/Build 6.0.388.0 from Agilent Technologies was used.

The standard for the BTX quantification was Absolute standard cat. No. 33003, 2000 µg/mL. Calibration curves were achieved for each analyte, from 2 ppm, to 200 ppm. Calibration curve levels were obtained from ten concentration levels from the BTX compounds, achieving curves with the values of 2, 4, 10, 20, 30, 40, 50, 100, 150, 200 ppm. Then after, a 5µL aliquot was taken of each one in a Tenax tube, resulting quantities of 20, 40, 100, 200, 300, 400, 500, 1000, 1500, 2000 ng. R^2 values ≥ 0.99, and at least six points were the acceptation criteria. Analytes were monitored in SCAN and SIM modes. Non quantified analytes found under the calibration curve were identified and quantified by deconvolution using toluene equivalents.

Analytes under the first level curve (less than 10.0045 ng) and a match or quality inferior to 70.

Analytes associated to the blanks and column bleed (siloxanes) were discarded.

3. Table 1SP, contains all the information related to BTX concentration data obtained from e-liquids and aerosols.

Table S1. BTX Concentrations of each sample.

	Benzene			Toluene			Xylenes		
Sample type / Sample number	E-liquids, water extraction µg/L	E-liquids, MeOH extraction µg/L	Aerosol µg/L	E-liquids, water extraction µg/L	E-liquids, MeOH extraction µg/L	Aerosol µg/L	E-liquids, water extraction µg/L	E-liquids, MeOH extraction µg/L	Aerosol µg/L
1	< 56.64	< 56.64	1 462.6	234.11	635.39	33 270.3	< 161.83	1183.20	3 735.3
2	< 56.64	208.85	577.7	< 73.84	< 73.84	1 010.4	182.97	7399.71	52 484.4
3	< 56.64	214.32	7 797.4	< 73.84	< 73.84	8 119.8	< 161.83	777.70	3 183.1
4	< 56.64	226.01	1 256.8	194.85	937.42	22 264.9	413.68	1139.19	1 952.7
5	< 56.64	180.40	577.2	87.68	304.99	3 532.2	< 161.83	1201.23	3 399.2
6	< 56.64	203.50	1 225.1	< 73.84	< 73.84	4 501.4	296.39	1722.36	1 163.9
7	< 56.64	279.33	2 158.6	< 73.84	669.64	4 234.9	17980.70	15429.80	165 555.8
8	72.01	227.29	1 326.6	1767.46	2100.84	130 618.7	1704.54	1157.07	16 827.4
9	< 56.64	238.19	2 104.3	< 73.84	390.87	6 598.9	215.31	980.78	4 015.2
10	< 56.64	< 56.64	517.1	1698.30	1430.37	15 652.1	1602.07	1824.81	10 171.9
11	< 56.64	416.34	1 168.4	1353.66	1011.78	19 334.0	621.39	1095.17	6 574.2
12	680.30	817.46	6 227.4	< 73.84	380.17	9 166.4	< 161.83	1012.71	2 891.8
13	< 56.64	189.95	1 508.6	< 73.84	278.21	115.6	276.48	< 161.83	799.0
14	123.96	< 56.64	2 169.6	< 73.84	2473.33	619.9	< 161.83	< 161.83	1 526.2
15	163.38	< 56.64	9 562.2	1811.54	< 73.84	39 176.6	3713.49	1383.85	18 365.5
16	< 56.64	< 56.64	1 755.1	< 73.84	< 73.84	39 924.4	202.81	1212.21	1 045.8
17	< 56.64	< 56.64	ND	< 73.84	< 73.84	1 007.4	162.60	904.29	1 767.3
18	< 56.64	199.58	1 733.12	2562.32	4355.03	102 795.4	1144.57	2507.66	11 831.4
19	< 56.64	< 56.64	1 242.5	< 73.84	< 73.84	2 005.1	166.42	< 161.83	1 319.9
20	< 56.64	< 56.64	3 023.9	2092.32	1398.77	64 564.6	2643.45	< 161.83	11 477.9

N.D. Non determined, quantification limit of each analyte is indicated as <, followed by the value.
